# A large scale classification of molecular fingerprints for the chemical space representation and SAR analysis

**DOI:** 10.1186/1758-2946-4-S1-P26

**Published:** 2012-05-01

**Authors:** Fabian López-Vallejo, Jacob Waddell, Austin B Yongye, Richard A Houghten, José L Medina-Franco

**Affiliations:** 1 Torrey Pines Institute for Molecular Studies, Port St. Lucie, Florida, 34987, USA

## 

Fingerprint-based structure representation has a broad range of applications including, but not limited to, diversity analysis, compound classification, chemical space visualization [1], activity landscape modelling and similarity searching. It has been shown that depending on the particular fingerprints used, the outcome of similarity searching [2] or activity landscapes [3] can be very different. Combining structure representations is a common practice to increase the performance of similarity searching [4]. Also, combining representations for activity landscape modelling has been proposed to generate robust descriptive SAR models [5]. However, the selection of fingerprints to be combined is not an easy task. As part of our efforts to select fingerprint representations to generate consensus representations of chemical space and activity landscapes [5,6] herein we discuss the results of a systematic comparison of more than 10 2D and 3D fingerprint representations in terms of performance in diversity analysis (as opposed to similarity searching). We employed more than 20 data sets from different sources relevant to drug discovery. In this work the widely used Tanimoto coefficient was employed. The approach presented here can be easily extended to other similarity measures, additional fingerprints and molecular databases. We also discuss the typical mean/median similarity values of selected fingerprints across databases from different sources.
